# Altered Effective Connectivity Network of the Amygdala in Social Anxiety Disorder: A Resting-State fMRI Study

**DOI:** 10.1371/journal.pone.0015238

**Published:** 2010-12-22

**Authors:** Wei Liao, Changjian Qiu, Claudio Gentili, Martin Walter, Zhengyong Pan, Jurong Ding, Wei Zhang, Qiyong Gong, Huafu Chen

**Affiliations:** 1 Key Laboratory for NeuroInformation of Ministry of Education, School of Life Science and Technology, University of Electronic Science and Technology of China, Chengdu, People's Republic of China; 2 Mental Health Center, West China Hospital of Sichuan University, Chengdu, People's Republic of China; 3 Unit of Clinical Psychology, AUO Pisa, Department of Psychiatry, Neurobiology, Pharmacology and Biotechnologies, University of Pisa, Pisa, Italy; 4 Clinical Affective Neuroimaging Laboratory, Department of Psychiatry, Otto von Guericke University, Magdeburg, Germany; 5 Huaxi MR Research Center (HMRRC), Department of Radiology, West China Hospital of Sichuan University, West China School of Medicine, Chengdu, People's Republic of China; The University of Melbourne, Australia

## Abstract

The amygdala is often found to be abnormally recruited in social anxiety disorder (SAD) patients. The question whether amygdala activation is primarily abnormal and affects other brain systems or whether it responds “normally” to an abnormal pattern of information conveyed by other brain structures remained unanswered. To address this question, we investigated a network of effective connectivity associated with the amygdala using Granger causality analysis on resting-state functional MRI data of 22 SAD patients and 21 healthy controls (HC). Implications of abnormal effective connectivity and clinical severity were investigated using the Liebowitz Social Anxiety Scale (LSAS). Decreased influence from inferior temporal gyrus (ITG) to amygdala was found in SAD, while bidirectional influences between amygdala and visual cortices were increased compared to HCs. Clinical relevance of decreased effective connectivity from ITG to amygdala was suggested by a negative correlation of LSAS avoidance scores and the value of Granger causality. Our study is the first to reveal a network of abnormal effective connectivity of core structures in SAD. This is in support of a disregulation in predescribed modules involved in affect control. The amygdala is placed in a central position of dysfunction characterized both by decreased regulatory influence of orbitofrontal cortex and increased crosstalk with visual cortex. The model which is proposed based on our results lends neurobiological support towards cognitive models considering disinhibition and an attentional bias towards negative stimuli as a core feature of the disorder.

## Introduction

Social anxiety disorder (SAD) [1], also termed social phobia, generally refers to an excessive fear and/or avoidance of a wide array of social situations [2]. Epidemiological survey conducted on general population has pointed out that lifetime prevalence of SAD ranges between 4.0% and 16% [3].

Recently an increasing number of neuroimaging researches have focused on characterizing brain circuits of SAD, allowing a comprehensive understanding of its anatomical and functional substrate [2], [4]–[5]. Specifically, findings from functional neuroimaging studies [for systemetic review see 2] indicated abnormalities in the neural circuitry including the amygdala, hippocampal and parahippocampal gyri, posterior insula, dorsal anterior cingulate cortex (ACC), ventrolateral prefrontal cortex (VLPFC) and temporal gyrus [6]–[12]. In any case, the most consistent finding in SAD studies through different experimental designs is the amygdala hyperactivity [2], [10], [13]–[16]. Furthermore, the amygdala hyperactivity in SAD might represent the functional correlation of emotional disregulation [17]–[19] and sustain threat-related processing bias [20].

Aside from investigations of the local brain activation, the role of the amygdala in SAD may be better understood if the amygdala is considered as a part of a more wide and complex dysfunctional network. For instance, impaired cortical and subcortical circuits connected with the amygdala were involved in the mediation of fear and anxiety [21]. Connectivity analysis using psychophysiological interactions demonstrated an abnormal connectivity between the amygdala and the VLPFC in adolescents with SAD [8]. Furthermore, influences of the fusiform gyrus on the amygdala in response to emotional faces have been explored in the non-clinical range of social anxiety [22].

One should, however, notice that all studies stated above based on task conditions. An extended understanding of SAD may still be achieved by detection of findings during the resting state that may not be present or may be masked during an activation paradigm [23]. Unlike traditional activation paradigms, resting-state studies observe intrinsic spontaneous fluctuations in the blood oxygen level-dependent (BOLD) functional magnetic resonance imaging (fMRI) signal in the absence of overt task performance or stimulation [24]–[26]. The spontaneous fluctuations demonstrate temporal coherences between distant brain structures and would functionally relate to through co-activations in response to task performance [27]. Consequently, resting-state fMRI studies were used for clinical applications towards understanding various neuropsychiatric disorders [for recent reviews see 28], [29]. A pioneering resting-state functional connectivity study has demonstrated an intra-amygdalar abnormality and engagement of a compensatory fronto-parietal executive control network of anxiety disorder [30]. It would allow to assess possible differences in the crosstalk among brain regions that could represent the baseline antecedent of abnormal activations in response to given tasks. Functional connectivity investigations documented that intrinsic resting-state brain activity is spatially organized in a set of specific coherent patterns and putatively corresponds to a specific brain function [31]–[35]. Interestingly, such patterns, namely resting-state networks (RSNs), recapitulate the functional architecture of somato-motor, visual, auditory, attention, language and memory networks that are commonly modulated during active behavioral task [36]–[38]. The RSNs are assumed to reflect the brain function of corresponding brain area; hence the abnormality of the resting brain activity can be used to imply the functional impairment/plastic of brain function. Among them, the default mode network (DMN) would be of considerable interest, as it consistently increased activity during rest compared to deactivations during cognitive tasks [25]. Findings from previous studies consistently implicate alterations of critical regions in DMN in patients with SAD [12], [23]. More recently, we showed a diffuse impact on widely distribution RSNs and selective changes of RSNs intrinsic functional connectivity in SAD [39].

The question whether amygdala activation is primarily abnormal and affects other brain systems or whether it responds “normally” to an abnormal pattern of information conveyed by other brain structures remained unanswered. To partially assess this topic, we aimed to evaluate altered directional connectivity patterns from and to the amygdala at rest in SAD as compared to HC and assess their possible relationship with clinical severity. This directed connectivity network was examined using Granger causality analysis (GCA) [40], which has been widely used for estimating prior and posterior prediction between BOLD fMRI time series [35], [41]–[49].

## Materials and Methods

### Participants

The first group was composed of 22 patients with SAD (all right-handed), who were recruited through the Mental Health Center of the Huaxi Hospital, Chengdu, China (**Table 1**). Diagnosis of SAD was determined by consensus between the two attending psychiatrists and a trained interviewer using the Structured Clinical Interview DSM-IV (SCID)-Patients Version. Subjects were excluded from the sample who presented any other psychiatric disorder according to structured clinical interview for DSM-IV Axis-I. Additionally, we excluded subjects who presented with a general medical condition. SAD patients were not under psychotherapy or psychiatric medications at the moment of the study, however, all patients underwent the psychotherapy and some of them underwent psychiatric medications after the study. The second group was composed of 22 age-, sex-, education matched healthy controls (HC) (all right-handed) who were recruited and screened using the SCID-Patients Version to confirm the current absence of psychiatric and neurological illness. Additionally, healthy controls were interviewed to confirm that there was no history of psychiatric illness among their first-degree relatives. Brain MR imaging (i.e. T1-weighted images) was inspected by an experienced neuroradiologist, and no gross abnormalities were observed in either group. All participants of the two groups were evaluated with the Spielberger State-Trait Anxiety Inventory (STAI-Y), Hamilton Anxiety Rating Scale (HAMA), Hamilton Depression Rating Scale (HAMD) and Liebowitz Social Anxiety Scale (LSAS). LSAS is one of the most widely used scale in SAD assessment. Its total score is composed by a fear factor (measuring the anxiety level of a given situation) and an avoiding one (related to the degree of avoidance of each situations). According to previous studies LSAS does not substitute a clinical interview for the diagnosis of social anxiety even if a score over 30 indicates a probable diagnosis of social anxiety while a score over 60 indicates a probable diagnosis of Generalized social anxiety [50].

**Table 1:** Psychological data or behavioral data.

	SAD^a^ (n = 22)	HC^a^ (n = 21)	SAD vs. HC	
			T Value	P Value
Gender (n:male/female)	16/6	15/6	-	0.9253^b^
Age (yrs)	22.55 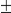 4.04	21.71 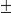 3.64	0.7076	0.4832
Education (yrs)	13.91 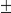 1.44	14 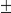 1.92	−0.1758	0.8613
Duration (mths)	52.18 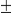 43.74	-	-	-
LSAS				
Total score	51.5 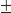 9.72	20.48 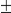 8.35	11.20	<0.0001
Fear factor	26.55 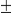 4.82	8.76 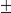 4.97	11.92	<0.0001
Avoidance factor	24.95 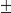 6.40	11.71 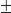 5.78	7.11	<0.0001
HAMD	8.45 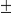 6.00	1.29 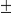 1.82	5.25	<0.001
HAMA	6.32 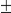 4.42	1.24 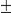 1.81	4.88	<0.001
STAI				
STAI-T	46.77 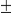 7.86	33.29 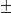 5.12	6.64	<0.0001
STAI-S				
Pre-scanning	40.95 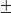 8.38	31.48 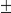 4.74	4.54	<0.001
Post-scanning	38.14 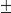 9.54	33.14 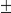 6.90	1.96	0.057

Abbreviations: SAD, social anxiety disorder; HC, healthy controls; LSAS, Liebowitz Social Anxiety Scale; HAMA, Hamilton Anxiety Rating Scale; HAMD, Hamilton Depression Rating Scale; STAI, Spielberger State-Trait Anxiety Inventory.

a Questionnaire data are given as mean ± standard deviation (SD).

b The *P* value was obtained by the Kruskal-Wallis test. The other 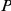 values were obtained by two-sample two-tailed *t*-test.

The present study was approved by the local Ethics Committee of Huaxi Hospital, Sichuan University, and written informed consents were obtained from all subjects. Some of the subjects (20 patients with SAD and 20 HCs) studies in the present work have also been used in our previous study [39], which assessed functional connectivity networks across the brain and thus regards a widely different and not overlapped analysis.

### Image Acquisition

Experiments were performed on a 3.0-T GE-Signa MRI scanner (EXCITE, General Electric, Milwaukee, USA) in Huaxi MR Research Center, Chengdu, China. Foam padding was used to minimize the head motion for all subjects. Functional images were acquired by a single-shot, gradient-recalled echo planar imaging (EPI) sequence (TR = 2000 ms, TE = 30 ms and flip angle  = 90°). Thirty transverse slices (FOV  = 24 cm, in-plane matrix  = 64×64, slice thickness  = 5 mm, without gap, voxel size  = 3.75×3.75×5), aligned along the anterior commissure-posterior commissure (AC-PC) line were acquired. For each subject, a total of 205 volumes were acquired, resulting in a total scan time of 410s. For the resting-state scans, subjects were instructed to rest with their eyes closed, without thinking of anything in particular and not falling asleep. After scanning, all participants reported that they were able to stay fully awake and have achieved the instruction to do nothing through all scanning. However, because such reports are potentially biased by participants wanting to appear compliant with instructions, it is not possible to know for certain what participants were thinking or feeling during scans. Moreover, a resting-state questionnaire [51] that is a robust and easy tool for exploration of the mental activities of participants during the resting state will be used in further work.

Subsequently, a high-resolution T1-weighted anatomical image was acquired in axial orientation using a 3D spoiled gradient recalled (SPGR) sequence (TR  = 8.5 ms, TE  = 3.4 ms, flip angle  = 12°, matrix size  = 512×512×156 and voxel size  = 0.47×0.47×1 mm^3^) for each subject.

### Data Preprocessing

Data preprocessing was carried out using the SPM2 software (http://www.fil.ion.ucl.ac.uk/spm). The first five volumes were discarded to ensure steady-state longitudinal magnetization. The remaining 200 volumes were slice-timing corrected relative to middle axial slice for the temporal difference in acquisition among different slices; and then volumes were registered to correct for head-motion during the scan. One of HCs was excluded from further analysis due to the head movement exceeded ±1.5 mm in translation and ±1.5° in rotation. The group differences in translation and rotation of head motion were also calculated according to the following formula [52]:
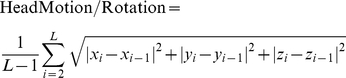
where 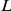 is the length of the time series (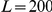 in this study), 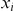,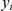 and 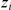 are translations/rotations at the *i*th time point in the 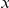,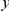 and 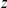 directions, respectively. The results showed that the two groups had no significant differences (two sample two-tailed *t*-test, 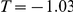, 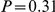 for translational motion and 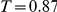, 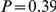 for rotational motion). The fMRI images were realigned with the corresponding T1-volume and warped into a standard stereotaxic space with a resolution of 3×3×3 mm^3^, using the Montreal Neurological Institute (MNI) echo-planar imaging template in SPM2. Finally, they were spatially smoothed by convolution with an isotropic Gaussian kernel (FWHM  = 8 mm).

### Effective Connectivity Analysis

The left and right amygdalas of the automated anatomical labeling (AAL) template were employed as the region of interest (ROI) for the effective connectivity analysis, respectively. ROIs were extracted using MarsBaR toolbox (http://marsbar.sourceforge.net). Averaged time series of these two ROIs were defined as the seed time series. Importantly, the rest time series of each voxel in whole brain and two seed time series were remove the possible spurious variances. Each time series was corrected for effect of head motion parameters by linear regression. Additionally, each time series was also corrected for white matter signal averaged from white matter (WM) and the cerebrospinal fluid signal averaged from cerebrospinal fluid (CSF) through linear regression according to previous resting-state fMRI studies [53]. To extract the time series for CSF and WM, subject-specific CSF and WM templates were created. These templates derived from the SPM template brain in which each subject's brain was warped. The residuals after regression constituted the voxel wise time series used for further analyses. A further band filter was applied (0.01∼0.08 Hz), for the purpose of reducing the effects of low-frequency drift and high-frequency noise. Finally, linear trends were removed from the data to eliminate the effect of gross signal drifts, which could be caused by scanner instabilities and/or gross physiological changes in the subject.

In the current study, Granger causality analysis was performed using an in-house program coded in MATLAB (The Mathworks, Natick, MA) in light of our previous studies [35], [41], [46]–[47]. More specifically, we used Granger causality to describe the effective connectivity between the ROI (bilateral amygdalae) and all other brain regions in each subject for both groups. The preprocessed mean time course of the ROI was defined as the seed time series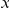, and the time series 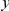 denotes the time series of all voxels in the brain. The linear direct influence of 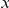 on 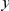 (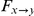), and the linear direct influence of 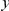 on (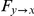) were calculated voxel by voxel in the brain [41]–[43], [46]–[47]. Thus, two Granger causality maps were generated based on the influence measures for each subjects. The order of the autoregressive model was set to 1 using the Schwartz criterion (SC). The coefficients of the models were calculated using a standard least squares optimization.

### Group-Level Analysis

Firstly, to assess the statistical significance of Granger causality results, we used the framework of the bootstrap methodology [54] to obtain the null distribution. This approach was widely employed in many fMRI studies to find the level of statistical significance without making assumptions on the underlying distribution of the data [41], [43]–[44], [46]. The permutation method was applied in the randomly sampling step by reshuffling the target time course 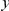 (or 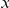 when calculating 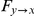). As 200∼5000 times of permutation is sufficient [54], 500 times were employed in this study.

For the group analysis on the effective connectivity (both left and right), mean values of the 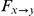 and 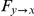 maps were calculated for each group. In total, eight group-level Granger causality maps were obtained, four for each direction and four for each group (i.e. the left amygdala with 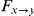 and 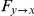 and the right amygdala with 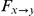 and 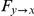 for the SAD group, as well as the HC group). When performing statistical tests on voxel level, the thresholds for significance thresholds were corrected for multiple comparisons using the false discovery rate (FDR) [55]. For further two groups comparison, a mask was created by considering significant voxels (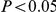, FDR corrected and a minimum cluster size of 10 voxels) either for SAD or for HC group. Finally, a direct comparison between the SAD and HC group was performed for each side and each direction. Only voxels covered by the masks created above were taken into account.

### Post-hoc Correlation Analysis

We performed a post-hoc Pearson correlation analysis in order to investigate the relationship between effective connectivity values and clinical severity. The voxels showing significantly different (increased or decreased) Granger influences between SAD and HC groups were extracted as a cluster. Mean Granger causality values within these cluster of each individual (SAD and HC group, separately) were correlated to the clinical severity measured using the Liebowitz social anxiety scale (including total score, fear factor and avoidance factor, respectively) and then thresholded at significance level of 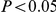, uncorrected.

## Results

We found significantly altered effective connectivity between the amygdala and temporal as well as prefrontal cortices, main effects are presented here, for a complete description of the areas showing an altered effective connectivity from or to the amygdala, please refer to the figures S1 and S2 and tables S1, S2, S3, S4, S5, S6, S7 and S8 in the supplementary material.

### Psychological Data or Behavioral Data

Group demographic characteristics and psychological scores are shown in **Table 1**. No significant differences were found between SAD patients and HCs in terms of gender, age, educational level and post-scanning of STAI-S. Compared with HCs, SAD patients showed significantly higher scores on the LSAS (including total score, fear factor and avoidance factor) assessment social anxiety symptom scales, and higher scores on the HAMD and HAMA, and higher levels of anxiety as assessed by the STAI-T and pre-scanning STAI-S.

### Effective Connectivity from the Left Amygdala

**Figure 1:**
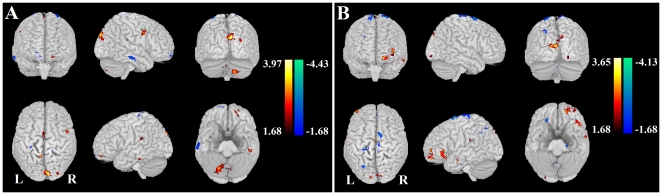
Altered effective connectivity from the amygdala. Altered effective connectivity from the left amygdala (A) and from the right amygdala (B) to other brain regions (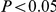, FDR corrected) when SAD compared to HC. The hot and cold colors indicate the brain regions that show significantly increased and decreased effective connectivity, respectively. The color scale represents T values.

Compared to HC, SAD patients showed significantly increased effective connectivity from the left amygdala to several brain regions which included the middle frontal cortex, temporal cortex, somato-motor and visual cortex and the cerebellum (**Fig. 1A**). Furthermore, decreased effective connectivity was found from the left amygdala to the left superior frontal gyrus (medial), and right middle temporal gyrus and the bilateral postcentral gyri (**Fig. 1A**, Table S1 and S2 and Figure S1).

### Effective Connectivity from the Right Amygdala

Compared to HC, SAD patients showed significant increased effective connectivity from the right amygdala to several brain regions that included the medial orbitofrontal gyrus (mOFG), temporal, occipital and limbic/paralimbic cortex (parahippocampus and hippocampus) (**Fig. 1B**). The results further revealed decreased effective connectivity from the right amygdala to the right superior frontal gyrus and hippocampus; and several regions in parietal lobe and cerebellum (**Fig. 1B**, Table S3 and S4 and Figure S1).

### Effective Connectivity to the Left Amygdala

**Figure 2:**
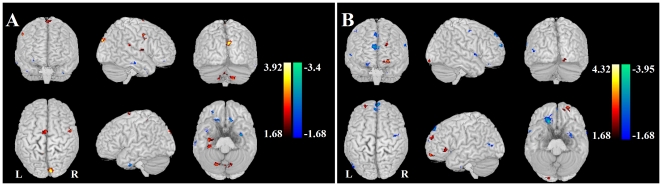
Altered effective connectivity from the other brain regions to the amygdala. Altered effective connectivity from the other brain regions to the left (A) and right amygdala (B) (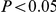, FDR corrected) when SAD compared to HC. The hot and cold colors indicate the brain regions that show significantly increased and decreased effective connectivity, respectively. The color scale represents T values.

SAD patients showed increased effective connectivity from other brain regions to the left amygdala, such as inform the default mode network (DMN) (namely, precuneus, middle cingulate gyrus), the visual cortex and the striatal cortex (putamina and pallidums) (**Fig. 2A**). Moreover, the superior frontal gyrus (medial), bilateral inferior temporal gyri (ITG) showed a significant decrease effective connectivity to the left amygdala in the SAD (**Fig. 2A**, Table S5 and S6 and Figure S2).

### Effective Connectivity to the Right Amygdala

SAD patients showed a significant stronger effective connectivity from the other brain regions to the right amygdala than in the HC, such as such as inform the DMN (namely, precuneus, middle and superior frontal gyri), the visual cortex and the striatal cortex (putamina and pallidums) (**Fig. 2B**). In addition, some brain regions showed a significant decrease effective connectivity to the right amygdala in the SAD, such as the frontal cortex and inferior temporal gyrus; and a few of regions in parietal lobe and subcortical cortices (**Fig. 2B**, Table S7 and S8 and Figure S2).

### Post-hoc Correlation between Clinical Scales

**Figure 3:**
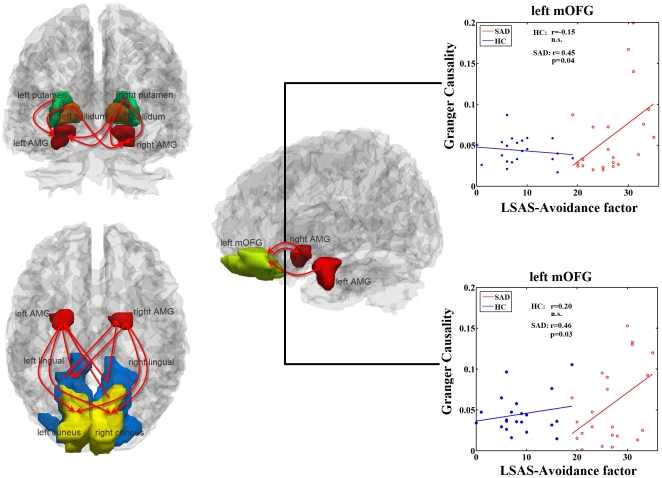
Regions showing increased effective connectivity. Increased directions are marked with red arrows in SAD compared to HC. Scatter plots showed correlations between effective connectivity in group level regions (see Figures 1 and 2) and avoidance factor in LSAS in SAD patients (red line and red open circles), and in HCs (blue line and blue solid circles), separately (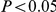). (AMG: amygdala, mOFG: medial orbitofrontal gyrus).

**Figure 4:**
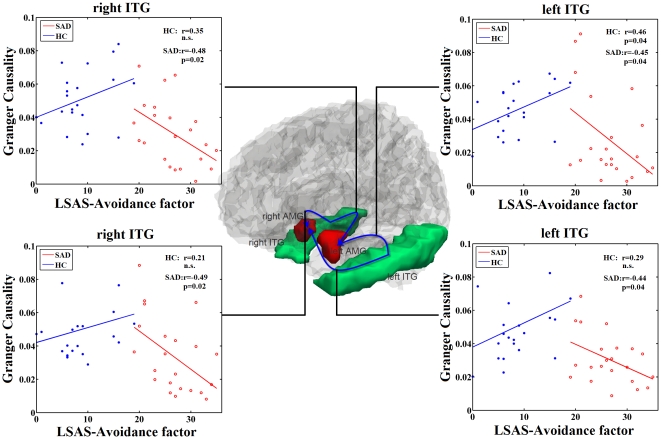
Regions showing decreased effective connectivity. Decreased directions are marked with blue arrows in SAD compared to HC. Scatter plots showed correlations between effective connectivity in group level regions (see Figures 1 and 2) and avoidance factor in LSAS in SAD patients (red line and red open circles), and in HCs (blue line and blue solid circles), separately (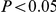). (AMG: amygdala, ITG: inferier temporal gyrus).

The mutual increased influences between the right amygdala the left mOFG were positively correlated with the avoidance factor of LSAS in SAD group, and were absent in the HC group (**Fig. 3**). Moreover, decreased influences from the left and right ITG to the bilateral amygdalae were negatively correlated with the avoidance factor of LSAS in SAD group, respectively (**Fig. 4**). As a matter of fact those influences just trended to positive correlation in HC group (for details see **Table 2**) (**Fig. 4**). The other brain regions with aberrant effective connectivity showed no significant correlation with LSAS.

**Table 2:** Relation between LSAS and Granger influence from and to the amygdale.

Directionality of influence	EC	Avoidance factor in LSAS	
	SAD vs. HC	SAD	HC
R AMG ← L mOFG	↑	0.46*	0.20
R AMG → L mOFG	↑	0.45*	0.15
R AMG ← R ITG	↓	−0.49*	0.21
R AMG ← L ITG	↓	−0.44*	0.29
L AMG ← R RTG	↓	−0.48*	0.35
L AMG ← L RTG	↓	−0.45*	0.46*

Abbreviations: AMG, amygdala gyrus; mOFG, medial orbitofrontal gyrus; ITG, inferior temporal gyrus; left; R, right; EC, effective connectivity; ↑, increased; ↓, decreased;

*denotes 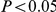, uncorrected.

## Discussion

To the best of our knowledge, this is the first study examined effective connectivity network associated with the amygdala of SAD patients during the resting state. We found an abnormal directionality of influence both from and to the amygdala in SAD patients using GCA. Moreover some of these abnormal connectivity patterns did not seem to rely directly on the degree of social anxiety on a continuum starting from HC to SAD, but are just present in SAD group. In any case they might represent a unique and distinctive characteristic of the pathological counterpart of the social anxiety phenomenon.

The current results revealed increased mutual influences between the medial orbitofrontal gyrus (mOFG) and amygdala in SAD patients. It has been demonstrated that these two regions are reciprocally connected with each other both functionally and anatomically [56]–[57]. As shown in literatures, the amygdala and orbitofrontal have been involved in the development of social cognition [58], and they may underly dysfunctional biases in emotion processing [59]. In SAD, orbitofrontal cortex hyporesponsivity and amygdala hyper-responsivity suggested emotional deregulations linked with failure to inhibit negative affect [17]. This evidence provides initial support that social anxiety might be associated with an altered balance of anygdala-prefrontal activity [59] and a consequent sustained threat-related processing bias [20]. Our findings were thus in line with these previous studies, and based on our results we suggested that increased interconnections between the amygdala and orbitofrontal cortex might attribute to sustained emotional deregulation between them. Furthermore, and as already stated, highly positive correlations between the Granger causality values and the avoidance factor of LSAS were only found in SAD patients, and were absent in the HC group.

The current data also showed differences comparing SAD to HC in mutual influences between the visual cortices (the bilateral lingual gyri, and cunei) and the amygdala. Recently, cortical pathways were declared in the social brain [60], in which the amygdala played a central role because of its importance in associating social stimuli with value [61]. In the so-called visual input pathways, visual information from a face is firstly processed in the inferior occipital gyrus and ultimately reaches the amygdala. Alternatively, modulated visual input can be obtained in the feedback from the amygdala to the inferior occipital gyrus. This pathway is sensitive to direct eye contact, especially in a situation involving threat [62]. Notably, patients with SAD usually blush rather than make eye contacts in social interactions and invariably experience intense emotional or physical symptoms, such as fear and, rapid heartbeat [1]. In addition, it was shown that SAD patients present a recognition bias with respect to threatening or critical facial expressions [63]–[66]. With the support of above findings, we suggest that increased interactions between the amygdala and occipital cortex might represent the neurobiological counterpart of their hypervigilance and hyperprosexia characteristic of patients with SAD [67]–[68]. It is also reported that social phobic processing of external social cues is biased in favor of responses and cues that can be interpreted negatively [69]. Also in the same study, Clark and Wells declared that SAD patients selectively retrieve negative information about themselves and their social performances and make negative evaluations of themselves and negative predictions about their future performance [69]. In this framework, the alteration of the information process might lead to an abnormally increased influence from the amygdala to the visual regions, which represents neurobiological underpinnings of the wariness and arousal. The directional characteristics of our findings exceed mere hyperactivity or connectivity but lend support to abnormal influences of amygdala on other brain structures.

In addition to those increased influence of amygdala, we also found decreased influences from the bilateral inferior temporal gyri (ITG) to the bilateral amygdalas. The ITG is among the last along the ventral visual pathway [70]. It is involved in object-processing [71] and its neurons are known to respond to face images [70]. Temporal cortex, together with the left amygdala and the right orbitofrontal cortex, was predominantly involved in the processing of negative expressions [72]. Furthermore, the amygdala received perceptual information from the occipital and temporal cortices and was involved in appraising the emotional significance of stimuli and guiding social decisions and social behavior [73]. In a previous study, enhanced cerebral blood flow in the left ITG has been detected in SAD patients [74]. Additionally, we found that the effective connectivity from the ITG to the amygdala was negatively correlated to the avoidance factor in SAD patients. In contrast, the positive correlation from the left ITG to the left amygdala was found in HC group. It is not possible to assess whether these differences are due to a “step” effect i.e., correlation with the degree of social anxiety becomes significant only over a certain threshold or if they represent a qualitative different pattern of connectivity. These abnormalities of the amygdala receiving information from the temporal cortex may enhance the bias of the processing of negative expressions in SAD patients.

Furthermore, we found increased influences from striatum (the bilateral pallidum and putamen) to the amygdala. These findings were consistent with one previous study which showed greater activities in striatal regions such as putamen and globus pallidus in social phobia patients [15]. This increased striatal regions activity was interpreted as that patients with social phobic react more with automatic emotion and use less cognitive processing.

While all these regional abnormalities in neuronal activity have been extensively described in prior studies, the network of abnormal influences of these core components of SAD is poorly understood. Exploring the communication of neuronal assemblies, however, is an important issue in human brain research and enjoys increasing attention also in the filed of non invasive neuroimaging. With the development of two recent-emerging state-of-the-art approaches, (i.e. GCA and dynamic causal modeling (DCM)), it is now possible to use fMRI data to extend the mechanism underlying the information processing in the brain [75]–[76]. Although both approaches estimate directed influences between brain systems using the temporal dynamics in the fMRI data, the fundamental and application of them are quite controversial [77]–[79]. Firstly, unlike DCM, GCA only requires the prespecification of ROIs and does not make any assumptions about the connections between them [80]. Therefore, in the current study, we only required to define the amygdala as ROI. Secondly, GCA imposes temporal precedence, which is intuitively linked to the concept that brain functional connectivity can be considered as directed (given the qualifications about hemodynamic latency variations above) [81]. Thirdly, being a stochastic model, GCA allows for the complexities involved in brain interactions [80].

We should note that there are some fundamental unanswered questions about the opportunity of applying GCA to fMRI data. First, the meaning of Granger influence at the neuronal level of resting state effective connectivity is not fully understood. Although a few resting state fMRI studies have revealed the causal influence among the resting state networks [35], [44]–[45], [82], it is believed that the causal influence is with the specific brain regions with which they interact. Second, it is noteworthy that the present findings related to connectivity pattern were observed during resting-state fMRI. Although several regions shown abnormal connectivity pattern in this sample were also found to be abnormally recruited in SAD patients during different emotional tasks, a direct relation of these two types of results remains difficult. Finally, since it is the first research on the directed influence between the amygdala and the other brain regions in SAD patients, a lenient significant threshold was employed for the correlations between effective connectivity and severity (i.e. the Granger causality value and the clinical scales). These analyses were exploratory in nature, and were to maximize sensitivity and to avoid missing significant findings with the use of a stricter threshold.

### Conclusions

In conclusion, we detected abnormalities in several amygdala-related pathways, which have been shown to be relevant for both expression and regulation of emotions. Our findings advance the knowledge on effective connectivity networks associated with the amygdala in SAD and more in general the neural rest circuitry underlying SAD. The role of RSNs in the neurobiology of mental functions is a matter of current debate. However in line with the hypothesis and experiments of recent researches [25]–[26], [33], [83], RSNs link to brain activity elicited during external-aimed tasks and maintain the brain ready to respond to external stimuli. Different baseline states could be, therefore, related and may explain different responses to the same stimuli. In this concern, we can speculate that decreased effective connectivity from the ITG to the amygdala might be the neurobiological correlate of the altered perceptive activity known as attentional bias in the cognitive models of SAD. The alteration of the information process might lead to an abnormally increased connectivity from the amygdala to the visual regions. Furthermore, increased interconnections between the amygdala and orbitofrontal cortex might represent the correlates of the cognitive-emotional disregulation. Our study adds the important notion of abnormal directional influence and further benefits from the absence of any potential bias of the task paradigm used for its investigation. However, further studies testing effective connectivity under emotional stress conditions are needed to confirm the present interpretation of our data and to better assess neurobiological correlates of attentional bias.

## Supporting Information

Figure S1
**Effective connectivity from the amygdala.** Effective connectivity from the left amygdala (left column), and from right amygdala (right column) to other brain regions (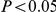, FDR corrected) in control group (top row), and in SAD group (bottom row). The warm color indicates the brain regions that show significantly effective connectivity.(TIF)

Figure S2
**Effective connectivity from the other brain regions to the amygdala.** Effective connectivity from the other brain regions to the left amygdale (left column) and the right amygdala (right column) (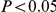, FDR corrected) in control group (top row), and in SAD group (bottom row). The warm color indicates the brain regions that show significantly effective connectivity.(TIF)

Table S1 Increased effective connectivity from the left amygdala to the other brain regions.
(DOC)

Table S2 Decreased effective connectivity from the left amygdala to the other brain regions.
(DOC)

Table S3 Increased effective connectivity from the right amygdala to the other brain regions.
(DOC)

Table S4 Decreased effective connectivity from the right amygdala to the other brain regions.
(DOC)

Table S5 Increased effective connectivity from the other brain regions to the left amygdale.
(DOC)

Table S6 Decreased effective connectivity from the other brain regions to the left amygdale.
(DOC)

Table S7 Increased effective connectivity from the other brain regions to the right amygdale.
(DOC)

Table S8 Decreased effective connectivity from the other brain regions to the right amygdale.
(DOC)
