# Supplementary figures and images for: Altered Effective Connectivity Network of the Amygdala in Social Anxiety Disorder: A Resting-State fMRI Study
Source: PLoS One. 2010 Dec 22;5(12):e15238. doi: 10.1371/journal.pone.0015238 (PMC3008679; doi:10.1371/journal.pone.0015238)

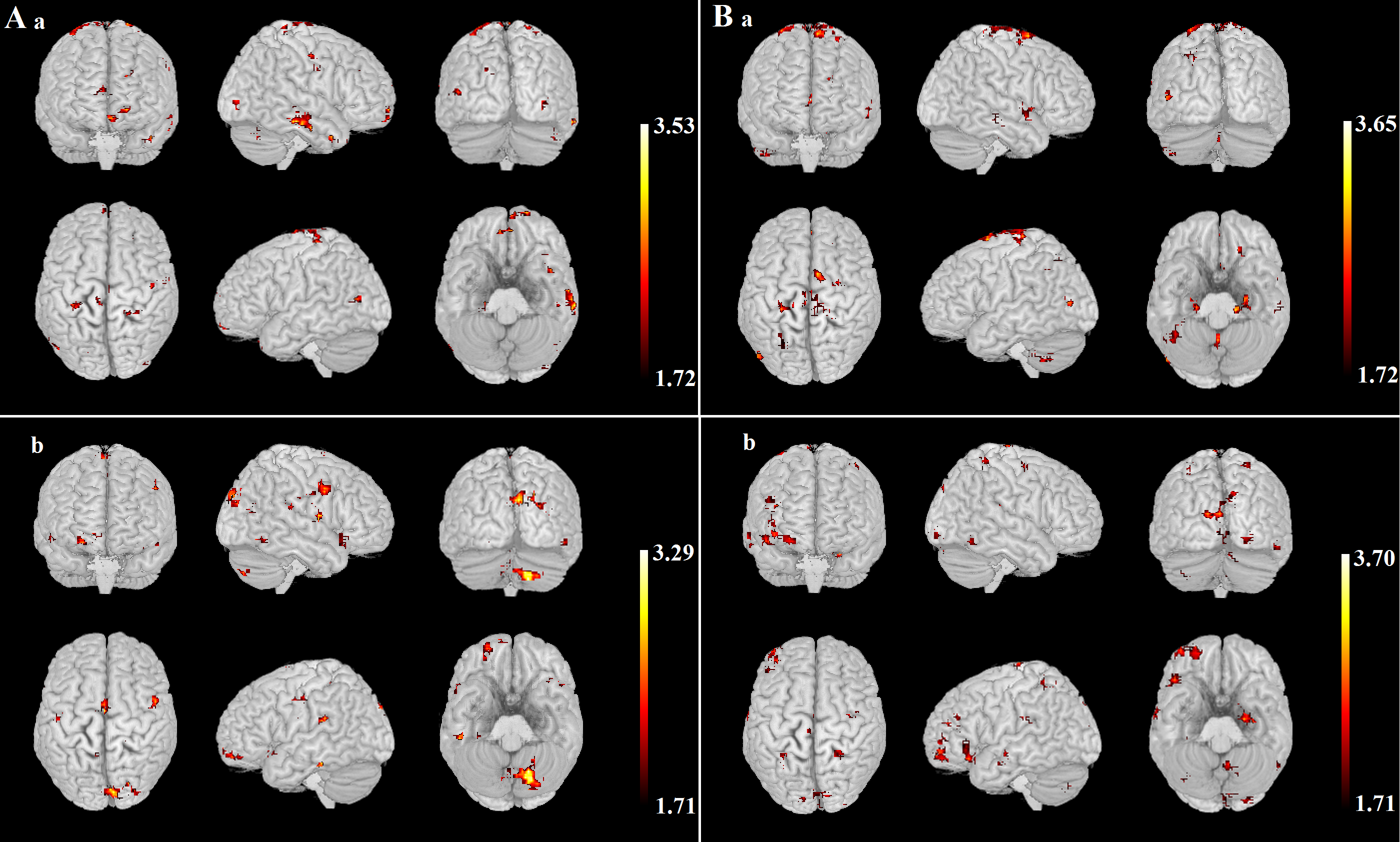

Supplement: Figure S1 — Effective connectivity from the amygdala. Effective connectivity from the left amygdala (left column), and from right amygdala (right column) to other brain regions (, FDR corrected) in control group (top row), and in SAD group (bottom row). The warm color indicates the brain regions that show significantly effective connectivity. (TIF) [file pone.0015238.s001.tif]

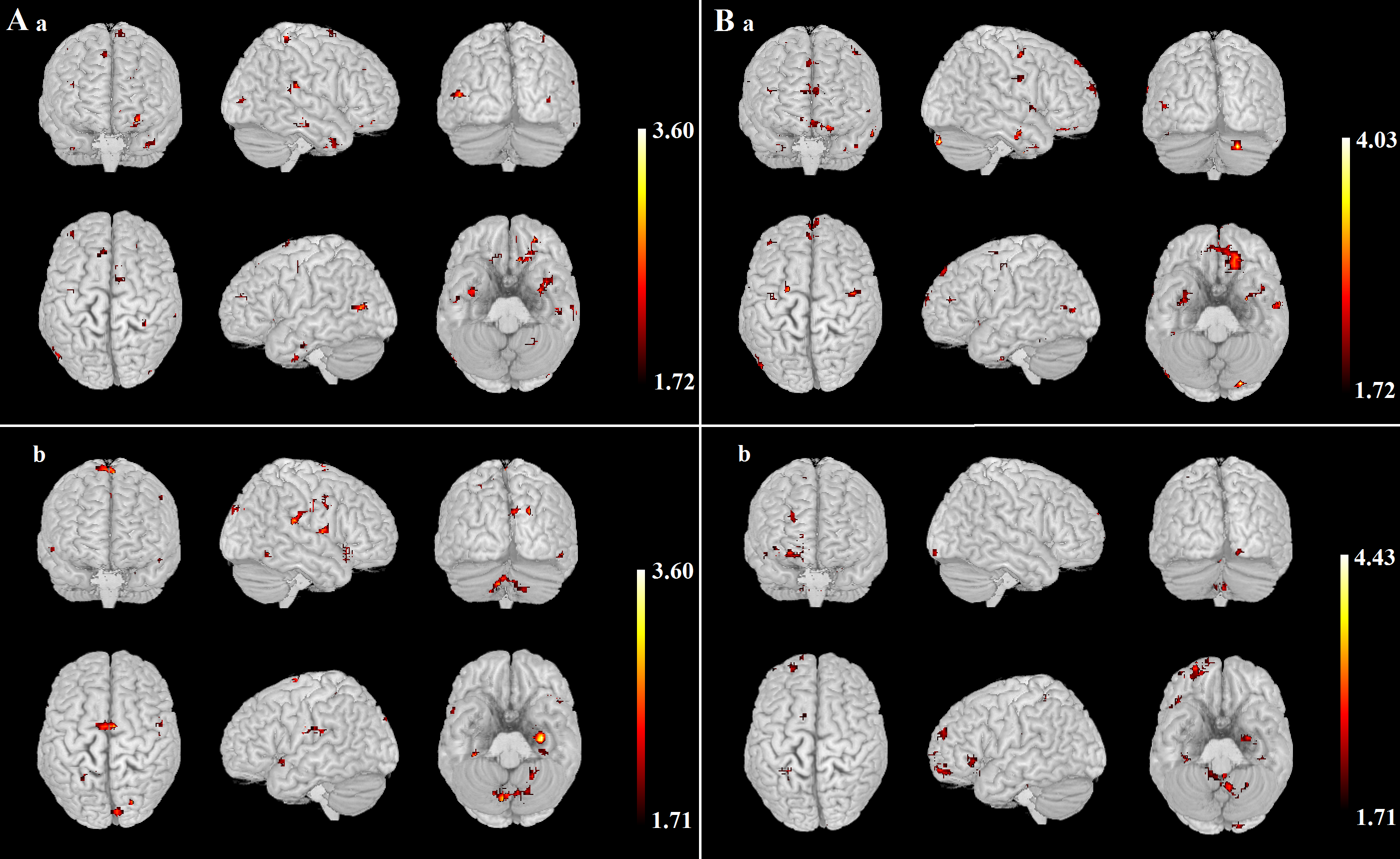

Supplement: Figure S2 — Effective connectivity from the other brain regions to the amygdala. Effective connectivity from the other brain regions to the left amygdale (left column) and the right amygdala (right column) (, FDR corrected) in control group (top row), and in SAD group (bottom row). The warm color indicates the brain regions that show significantly effective connectivity. (TIF) [file pone.0015238.s002.tif]
