# Supplementary material for: Altered Effective Connectivity Network of the Amygdala in Social Anxiety Disorder: A Resting-State fMRI Study
Source: PLoS One. 2010 Dec 22;5(12):e15238. doi: 10.1371/journal.pone.0015238 (PMC3008679; doi:10.1371/journal.pone.0015238)
Supplement: Table S1 — Increased effective connectivity from the left amygdala to the other brain regions. (DOC) [file pone.0015238.s003.doc]

**Table S1**

Increased effective connectivity from the left amygdala to the other brain regions

| Region name | Hem | voxels | MNI(x,y,z) | T value | BA |
| --- | --- | --- | --- | --- | --- |
| *Frontal* |  |  |  |  |  |
| Middle frontal gyrus, orbital | L | 14 | -18,54-15 | 3.1426 | 11 |
| *Temporal* |  |  |  |  |  |
| Inferior temporal gyrus | L | 11 | -51,-33,-21 | 3.2223 | 20 |
|  | R | 13 | 54,-54,-12 | 2.6131 | 20,37 |
| *Occipital* |  |  |  |  |  |
| Cuneus | L | 19 | 3,-84,30 | 2.9611 | 18,19 |
|  | R | 38 | 9,-87,30 | 3.9743 | 18,19 |
| Calcarine fissure | L | 19 | -9,-69,12 | 2.8865 | 17 |
| Lingual gyrus | L | 10 | -21,-78,-12 | 1.9686 | 17,18 |
| Superior occipital gyrus | R | 14 | 12,-93,36 | 2.7819 | 18,19 |
| Middle occipital gyrus | R | 12 | 30,-90,24 | 2.7397 | 19 |
| *Parietal-(pre)Motor* |  |  |  |  |  |
| Supplementary motor area | L | 27 | -12,-9,69 | 2.874 | 6 |
| Precentral gyrus | R | 26 | 51,3,36 | 3.1402 | 3,6,44 |
| Precuneus | L | 23 | -6,-51,60 | 3.8611 | 4,5 |
| *Cerebelum* |  |  |  |  |  |
| Cerebelum_7b | R | 15 | 18,-78,-45 | 2.944 | - |
| Cerebelum_8 | R | 56 | 18,-72,-48 | 3.274 | - |
| Cerebelum_Crus2 | L | 11 | -3,-78,-36 | 2.6945 | - |
|  | R | 14 | 21,-78,-45 | 2.4744 | - |

Hem, hemisphere; BA, Brodmann’s area; MNI (x,y,z), coordinates of primary peak locations in the space of Montreal Neurological Institute (MNI).
