# Supplementary material for: Altered Effective Connectivity Network of the Amygdala in Social Anxiety Disorder: A Resting-State fMRI Study
Source: PLoS One. 2010 Dec 22;5(12):e15238. doi: 10.1371/journal.pone.0015238 (PMC3008679; doi:10.1371/journal.pone.0015238)
Supplement: Table S2 — Decreased effective connectivity from the left amygdala to the other brain regions. (DOC) [file pone.0015238.s004.doc]

**Table S2**

Decreased effective connectivity from the left amygdala to the other brain regions

| Region name | Hem | voxels | MNI(x,y,z) | T value | BA |
| --- | --- | --- | --- | --- | --- |
| *Frontal* |  |  |  |  |  |
| Superior frontal gyrus, medial | L | 14 | -6,51,15 | -2.3726 | 9,10,32 |
| *Temporal* |  |  |  |  |  |
| Middle temporal gyrus | R | 31 | 66,-24,-15 | -2.6075 | 20,21,22 |
| *Parietal-(pre)Motor* |  |  |  |  |  |
| Precentral gyrus | L | 12 | -30,-30,75 | -2.718 | 4 |
|  | R | 15 | 21,-39,81 | -3.1358 | 1,3,4 |

Hem, hemisphere; BA, Brodmann’s area; MNI (x,y,z), coordinates of primary peak locations in the space of Montreal Neurological Institute (MNI).
