# Supplementary material for: Altered Effective Connectivity Network of the Amygdala in Social Anxiety Disorder: A Resting-State fMRI Study
Source: PLoS One. 2010 Dec 22;5(12):e15238. doi: 10.1371/journal.pone.0015238 (PMC3008679; doi:10.1371/journal.pone.0015238)
Supplement: Table S3 — Increased effective connectivity from the right amygdala to the other brain regions. (DOC) [file pone.0015238.s005.doc]

**Table S3**

Increased effective connectivity from the right amygdala to the other brain regions

| Region name | Hem | voxels | MNI(x,y,z) | T value | BA |
| --- | --- | --- | --- | --- | --- |
| *Frontal* |  |  |  |  |  |
| Middle frontal gyrus, orbital | L | 45 | -18,54,-15 | 3.6495 | 10,11,46,47 |
| Inferior frontal gyrus, orbital | L | 42 | -48,24,-12 | 2.6908 | 38,47 |
| Inferior frontal gyrus, triangular | L | 18 | -42,27,3 | 2.9671 | 45,47 |
| Middle frontal gyrus | L | 14 | -39,54,3 | 2.9931 | 10,45,46 |
| *Temporal* |  |  |  |  |  |
| Middle temporal gyrus | L | 10 | -63,-3,-21 | 2.8959 | 21 |
| ParaHippocampal | R | 15 | 24,-3,-27 | 2.5936 | 20,28,30,35,36 |
| Hippocampus | R | 11 | 24,-9,-18 | 2.7048 | 20,35,36 |
| *Occipital* |  |  |  |  |  |
| Calcarine fissure | L | 43 | -3,-81,15 | 3.36 | 17,18,19 |
|  | R | 10 | 15,-78,12 | 2.607 | 17,18,19 |
| Lingual gyrus | L | 13 | -15,-66,0 | 2.7546 | 17,18,19,37 |
| Cuneus | L | 43 | -3,-81,18 | 3.617 | 17,18,19 |
|  | R | 16 | 12,-87,39 | 2.6541 | 18,19 |
| Superior occipital gyrus | L | 18 | -12,-87,18 | 3.1672 | 17,18,19 |
| *Parietal-(pre)Motor* |  |  |  |  |  |
| SupraMarginal gyrus | L | 13 | -45,-36,24 | 3.2939 | 22,48 |
| Supplementary motor area | L | 12 | -3,-6,54 | 3.5253 | 6 |

Hem, hemisphere; BA, Brodmann’s area; MNI (x,y,z), coordinates of primary peak locations in the space of Montreal Neurological Institute (MNI).
