# Supplementary material for: Altered Effective Connectivity Network of the Amygdala in Social Anxiety Disorder: A Resting-State fMRI Study
Source: PLoS One. 2010 Dec 22;5(12):e15238. doi: 10.1371/journal.pone.0015238 (PMC3008679; doi:10.1371/journal.pone.0015238)
Supplement: Table S4 — Decreased effective connectivity from the right amygdala to the other brain regions. (DOC) [file pone.0015238.s006.doc]

**Table S4**

Decreased effective connectivity from the right amygdala to the other brain regions

| Region name | Hem | voxels | MNI(x,y,z) | T value | BA |
| --- | --- | --- | --- | --- | --- |
| *Frontal* |  |  |  |  |  |
| Superior frontal gyrus | R | 15 | 15,3,75 | -2.4359 | 6,9,32 |
| *Temporal* |  |  |  |  |  |
| Inferior parietal gyrus | L | 27 | -33,-54,39 | -4.124 | 7,40 |
| Superior parietal gyrus | L | 15 | -30,-66,51 | -2.8234 | 7 |
| Postcentral gyrus | L | 23 | -24,-27,78 | -2.6095 | 4 |
| Precentral | L | 17 | -24,-21,78 | -2.6398 | 6 |
| Supplementary motor area | R | 23 | 12,6,75 | -3.5869 | 6 |
| Paracentral lobule | R | 15 | 6,-18,81 | -2.1113 | 4 |
| *Cerebelum* |  |  |  |  |  |
| Cerebelum_4_5 | L | 21 | -12,-42,-24 | -2.4397 | 19,27,30 |
| Vermis_9 |  | 11 | -3,-54,-30 | -4.1325 | - |

Hem, hemisphere; BA, Brodmann’s area; MNI (x,y,z), coordinates of primary peak locations in the space of Montreal Neurological Institute (MNI).
