# Supplementary material for: Altered Effective Connectivity Network of the Amygdala in Social Anxiety Disorder: A Resting-State fMRI Study
Source: PLoS One. 2010 Dec 22;5(12):e15238. doi: 10.1371/journal.pone.0015238 (PMC3008679; doi:10.1371/journal.pone.0015238)
Supplement: Table S5 — Increased effective connectivity from the other brain regions to the left amygdale. (DOC) [file pone.0015238.s007.doc]

**Table S5**

Increased effective connectivity from the other brain regions to the left amygdala

| Region name | Hem | voxels | MNI(x,y,z) | T value | BA |
| --- | --- | --- | --- | --- | --- |
| *Temporal* |  |  |  |  |  |
| ParaHippocampal | R | 40 | 36,-15,-30 | 2.921 | 20,30,35,36,37 |
| Fusiform gyrus | R | 39 | 36,-18,-30 | 3.0023 | 19,20,30,37 |
| *Occipital* |  |  |  |  |  |
| Lingual gyrus | R | 10 | 24,-66,-3 | 2.3598 | 18,19,37 |
| Cuneus | R | 24 | 9,-90,33 | 3.6451 | 18,19 |
| *Parietal-(pre)Motor* |  |  |  |  |  |
| Precuneus | L | 16 | -9,-51,60 | 2.8704 | 4,5,19,37 |
| Precentral gyrus | R | 28 | 51,6,36 | 3.1131 | 4,6,44 |
| Median cingulate gyrus | R | 13 | 9,-9,42 | 3.6963 | 23,24 |
| Supplementary motor area | L | 31 | 0,0,72 | 2.6582 | 6 |
| *Subcortical* |  |  |  |  |  |
| Putamen | L | 24 | -18,9,3 | 3.7195 | 11,25,48 |
|  | R | 11 | 18,6,-6 | 2.5302 | 25,48 |
| Pallidum | L | 21 | -18,6,3 | 3.4759 | 25,48 |
|  | R | 28 | 15,3,-3 | 3.6309 | 48 |
| *Cerebelum* |  |  |  |  |  |
| Cerebelum_8 | L | 14 | -15,-69,-51 | 2.6189 | - |
|  | R | 26 | 18,-72,-48 | 2.8289 | - |

Hem, hemisphere; BA, Brodmann’s area; MNI (x,y,z), coordinates of primary peak locations in the space of Montreal Neurological Institute (MNI).
