# Supplementary material for: Altered Effective Connectivity Network of the Amygdala in Social Anxiety Disorder: A Resting-State fMRI Study
Source: PLoS One. 2010 Dec 22;5(12):e15238. doi: 10.1371/journal.pone.0015238 (PMC3008679; doi:10.1371/journal.pone.0015238)
Supplement: Table S6 — Decreased effective connectivity from the other brain regions to the left amygdale. (DOC) [file pone.0015238.s008.doc]

**Table S6**

Decreased effective connectivity from the other brain regions to the left amygdala

| Region name | Hem | voxels | MNI(x,y,z) | T value | BA |
| --- | --- | --- | --- | --- | --- |
| *Frontal* |  |  |  |  |  |
| Superior frontal gyrus, medial | L | 10 | 0,48,45 | -2.7247 | 8,9,10 |
|  |  |  |  |  |  |
| *Temporal* |  |  |  |  |  |
| Inferior temporal gyrus | L | 21 | -36,-6,-39 | -3.1253 | 20,36 |
|  | R | 16 | 63,-21,-21 | -2.4142 | 20,37 |
| *Parietal-(pre)Motor* |  |  |  |  |  |
| Precuneus | L | 10 | -6,-48,15 | -2.8049 | 17,29,30 |
| *Cerebelum* |  |  |  |  |  |
| Vermis_3 |  | 12 | 6,-36,-6 | -2.4736 | 27,30 |

Hem, hemisphere; BA, Brodmann’s area; MNI (x,y,z), coordinates of primary peak locations in the space of Montreal Neurological Institute (MNI).
