# Supplementary material for: Altered Effective Connectivity Network of the Amygdala in Social Anxiety Disorder: A Resting-State fMRI Study
Source: PLoS One. 2010 Dec 22;5(12):e15238. doi: 10.1371/journal.pone.0015238 (PMC3008679; doi:10.1371/journal.pone.0015238)
Supplement: Table S7 — Increased effective connectivity from the other brain regions to the right amygdale. (DOC) [file pone.0015238.s009.doc]

**Table S7**

Increased effective connectivity from the other brain regions to the right amygdala

| Region name | Hem | voxels | MNI(x,y,z) | T value | BA |
| --- | --- | --- | --- | --- | --- |
| *Frontal* |  |  |  |  |  |
| Middle frontal gyrus, orbital | L | 23 | -27,60,-12 | 3.2927 | 11,46,47 |
| Inferior frontal gyrus, orbital | L | 11 | -42,27,-3 | 2.6153 | 38,47 |
| Superior frontal gyrus | L | 18 | -24,60,27 | 2.8062 | 10,46 |
| Inferior frontal gyrus, triangular | L | 12 | -45,24,0 | 2.6476 | 47 |
| *Temporal* |  |  |  |  |  |
| ParaHippocampal | L | 14 | -21,-9,-30 | 2.2119 | 27,36,37 |
| Fusiform gyrus | L | 26 | -27,-54,-9 | 2.9766 | 19,20,37 |
| *Occipital* |  |  |  |  |  |
| Lingual gyrus | L | 57 | -24,-51,-3 | 3.461 | 18,19,30,37 |
|  | R | 23 | 15,-96,-15 | 2.9184 | 18,19,27,37 |
| *Parietal-(pre)Motor* |  |  |  |  |  |
| Precuneus | L | 11 | -21,-48,0 | 3.3443 | 19,37 |
| *Subcortical* |  |  |  |  |  |
| Pallidum | L | 26 | -15,3,0 | 3.2209 | 48 |
|  | R | 34 | 15,0,-3 | 4.3194 | 48 |
| Thalamus | L | 20 | -15,-18,15 | 3.1345 | 27 |
|  | R | 31 | 18,-15,6 | 2.8491 | - |
| Putamen | L | 10 | -18,9,3 | 3.1345 | 48 |
| *Cerebelum* |  |  |  |  |  |
| Cerebelum_8 | R | 16 | 12,-63,-36 | 2.3188 | - |
| Cerebelum_9 | L | 13 | -6,-45,-51 | 2.8926 | - |
|  | R | 12 | 6,-45,-51 | 2.4817 | - |
| Cerebelum_4_5 | L | 10 | -9,-57,-6 | 2.8063 | 18 |

Hem, hemisphere; BA, Brodmann’s area; MNI (x,y,z), coordinates of primary peak locations in the space of Montreal Neurological Institute (MNI).
