# Supplementary material for: Altered Effective Connectivity Network of the Amygdala in Social Anxiety Disorder: A Resting-State fMRI Study
Source: PLoS One. 2010 Dec 22;5(12):e15238. doi: 10.1371/journal.pone.0015238 (PMC3008679; doi:10.1371/journal.pone.0015238)
Supplement: Table S8 — Decreased effective connectivity from the other brain regions to the right amygdale. (DOC) [file pone.0015238.s010.doc]

**Table S8**

Decreased effective connectivity from the other brain regions to the right amygdala

| Region name | Hem | voxels | MNI(x,y,z) | T value | BA |
| --- | --- | --- | --- | --- | --- |
| *Frontal* |  |  |  |  |  |
| Superior frontal gyrus, orbital | R | 40 | 15,30,-18 | -3.5912 | 11 |
| Superior frontal gyrus, medial | L | 52 | -3,54,18 | -3.5168 | 9,10,32 |
|  | R | 33 | 6,60,21 | -3.2499 | 9,10,32 |
| Superior frontal gyrus | L | 12 | -24,0,66 | -2.8598 | 6 |
| Rectus | R | 20 | 15,30,-18 | -3.5912 | 11 |
| *Temporal* |  |  |  |  |  |
| Inferior temporal gyrus | L | 14 | -33,0,-39 | -3.0285 | 20,36,37 |
|  | R | 13 | 45,-45,-12 | -3.1285 | 20,36,37 |
| Fusiform gyrus | L | 10 | -33,-3,-39 | -2.5442 | 20,36,37 |
| *Parietal-(pre)Motor* |  |  |  |  |  |
| Precentral gyrus | R | 10 | 42,-9,51 | -2.4841 | 4,6 |
| *Subcortical* |  |  |  |  |  |
| Caudate nucleus | R | 19 | 15,24,0 | -2.3492 | 11,25 |

Hem, hemisphere; BA, Brodmann’s area; MNI (x,y,z), coordinates of primary peak locations in the space of Montreal Neurological Institute (MNI).
